# Global assessment of flood and storm extremes with increased temperatures

**DOI:** 10.1038/s41598-017-08481-1

**Published:** 2017-08-11

**Authors:** Conrad Wasko, Ashish Sharma

**Affiliations:** School of Civil and Environmental Engineering, University of New South Wales, Sydney, 2052 Australia

## Abstract

There is overwhelming consensus that the intensity of heavy precipitation events is increasing in a warming world. It is generally expected such increases will translate to a corresponding increase in flooding. Here, using global data sets for non-urban catchments, we investigate the sensitivity of extreme daily precipitation and streamflow to changes in daily temperature. We find little evidence to suggest that increases in heavy rainfall events at higher temperatures result in similar increases in streamflow, with most regions throughout the world showing decreased streamflow with higher temperatures. To understand why this is the case, we assess the impact of the size of the catchment and the rarity of the event. As the precipitation event becomes more extreme and the catchment size becomes smaller, characteristics such as the initial moisture in the catchment become less relevant, leading to a more consistent response of precipitation and streamflow extremes to temperature increase. Our results indicate that only in the most extreme cases, for smaller catchments, do increases in precipitation at higher temperatures correspond to increases in streamflow.

## Introduction

The cost of annual global flood damages was estimated at over $50 billion in 2013 alone^1^ and is expected to more than double within the next twenty years^2^ from increasing populations and intensification of precipitation extremes^3–5^. As long-lasting or intense precipitation is often the main cause of flooding^6^, precipitation trends have been used to suggest future increases in flooding^7^. However, historical flood events do not correspond well to extreme precipitation anomalies^7–9^. There is little observational evidence that flood magnitudes have increased^10–12^. In fact, observational records present more evidence for a decrease in annual flood maxima^13–15^, despite increases in precipitation being well documented^16–18^.

While there have been notable improvements in modelling precipitation extremes^19^, the representation of small scale precipitation formation processes in global and regional climate models remains limited, resulting in poor local precipitation estimates^20^. Hence, historical relationships of precipitation and temperature, termed scaling, are frequently used to evaluate future changes to precipiation^4, 21, 22^. As the Clausius-Clapeyron relationship states there is a 7%/°C increase in the maximum amount of low-level moisture in the atmosphere, assuming invariant relative humidity, there exists a physical argument for the possible increase in the maximum amount of precipitation that may occur under a global warming scenario^23, 24^. Global studies of the sensitivity of precipitation to temperature show significant variability due to local climate and deviation from the theoretical sensitivity^25, 26^. With controversy due to aggregating over a wide range of climate dynamics^27–30^, the use of scaling relationships is justified by historical increases in precipitation matching observed sensitivities^16, 21, 31^. Despite extensive use of precipitation scaling relationships to infer changes to local flooding^21, 22, 24, 32, 33^, the consistency between the scaling relationship for precipitation and streamflow with temperature has never been verified.

Here, we investigate whether streamflow-temperature sensitivity exhibits patterns that are similar to precipitation-temperature sensitivity. Through a range of analyses we find that streamflow-temperature sensitivity is largely negative while precipitation-temperature sensitivity is positive, implying that while rising temperatures lead to an increase in the incident precipitation, a decrease in the streamflow is observed, except in the rarest of cases or when catchments are small.

## Results

### Global relationship of streamflow and precipitation with temperature

**Figure 1:**
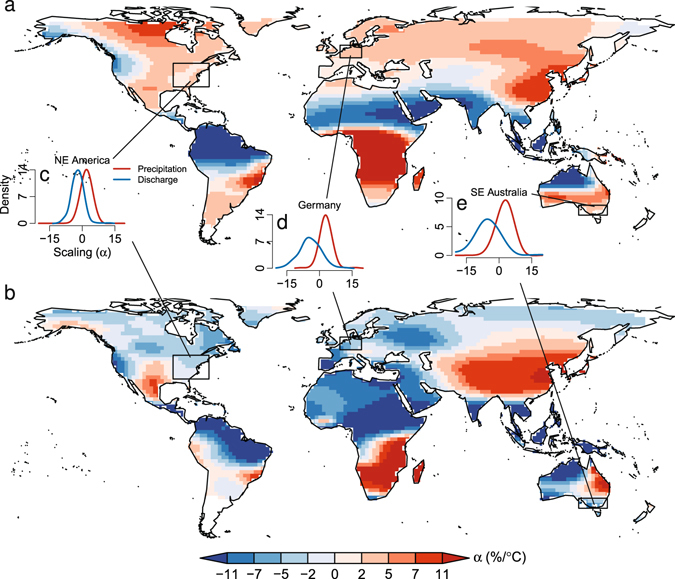
Precipitation and streamflow scaling with temperature for the 99^th^ percentile. (**a**) Precipitation scaling. (**b**) Streamflow scaling. (**c**) Probability density of scaling coefficients for north east America. (**d**) Probability density of scaling coefficients for Germany. (**e**) Probability density of scaling coefficients for south east Australia. Scaling has been calculated using quantile regression and interpolated using a thin plate smoothing spline. This figure was created using the ‘maps’^59^ and ‘mapdata’^60^ packages in the statistical software ‘R’^61^.

We begin by calculating the sensitivity of daily precipitation and daily streamflow with temperature. For each station, precipitation events and streamflow events were identified separately and the peak precipitation and peak streamflow within each event selected for analysis. Precipitation events were identified where precipitation was separated by five days of zero rainfall, streamflow events were selected on a basis of the peaks being separated by more than seven days. Where the separation was less than seven days, the peaks were considered as part of the same streamflow event. The largest peak from each precipitation and streamflow event was then chosen and matched to its coincident surface temperature resulting in a set of precipitation-temperature and streamflow-temperature pairs for each gauge site. The surface temperature was obtained from a 1° × 1° global gridded data set with each precipitation and streamflow gauge matched by the shortest distance to the grid point centre. Following similar studies, at each gauge location, the 99^th^ percentile scaling of the precipitation-temperature and streamflow-temperature data pairs was calculated using quantile regression^34, 35^. The scaling with temperature was calculated independently for the precipitation and streamflow. The scaling, interpolated over land, and smoothed using a thin plate smoothing spline is presented in Fig. 1.

Precipitation scaling has a strong trend with latitude. It is positive in the subtropics and temperate regions and negative in the tropics, with the exception of local regions in the western U.S. and Pacific Islands (Fig. 1a). The streamflow scaling (Fig. 1b), however, has no apparent trend with latitude and is predominately negative. Regions throughout Europe, North America, and southern Australia scale negatively for streamflow but positively for precipitation. Inserts of the probability density functions of the precipitation and streamflow scaling coefficients for NE America (Fig. 1c), Germany (Fig. 1d) and SE Australia (Fig. 1e) show a greater scaling for precipitation with temperature than for streamflow. At the 99^th^ percentile, despite scaling relationships suggesting increases in precipitation at higher temperatures, the streamflow scaling is negative.

### The role of hydrologic losses

The general assumption that an increase in precipitation extremes with higher temperatures translates to an increase in the extreme flows is not substantiated. To understand this result a discussion is required. In a setting where all the precipitation is converted into overland streamflow as a result of the precipitation event, a change in precipitation will have a one-to-one correspondence to a change in streamflow. This however is rarely the case. As precipitation falls to the ground it will not all directly contribute to the streamflow. The precipitation not directly contributing to streamflow is termed in hydrology as ‘abstractions’ or ‘losses’^36^. Sources of loss include transmission to the subsurface, storage in surface depressions, and, to a lesser extent, evaporation and evapotranspiration. If a proportion of the precipitation causing a streamflow peak immediately after a storm is ‘lost’, volumetrically the streamflow is less than the precipitation. However, as scaling relationships investigate the change with higher temperatures, if the loss remains constant, an increase in precipitation should result in a similar increase in streamflow. Hence, why the streamflow scaling differs from the precipitation scaling is that the precipitation ‘lost’ is not constant. Accounting for all these sources of moisture loss is complicated, so we seek an alternative.

### Consideration of precipitation intensity

As the precipitation event becomes more extreme, the precipitation intensity increases, and the effect of any depression storage and transmission to the subsurface on the resultant flood peak decreases^37^. As there is insufficient data at point locations to investigate more extreme percentiles, the point based data presented in Fig. 1 is aggregated over homogenous regions after standardising by the mean to remove catchment scale differences. To extrapolate the scaling to the 99.99^th^ percentile the data is binned using 2 °C temperature bins and the scaling calculated by fitting a linear regression to computed extreme percentiles in each bin^22^.

**Figure 2:**
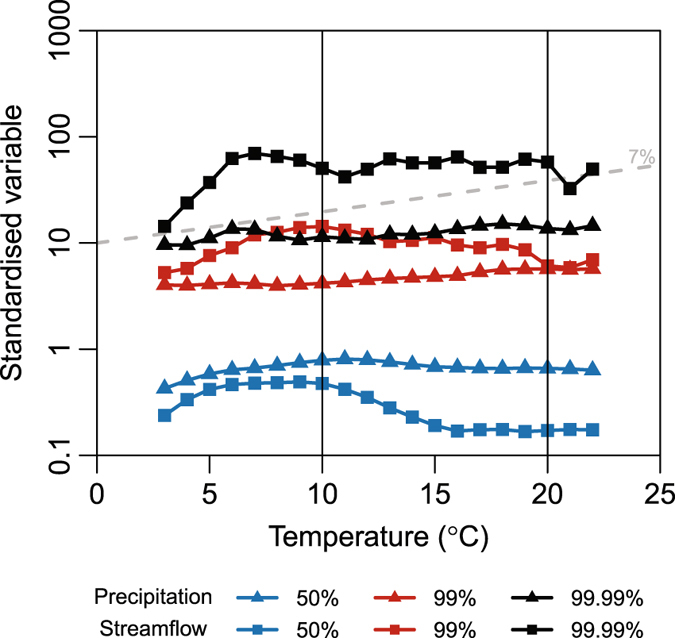
Precipitation and streamflow percentiles with temperature for South East Australia. Precipitation and streamflow aggregations have been divided by the mean value at that gauge. The 50^th^ and 99^th^ percentile were calculated empirically from the data using 2 °C temperature bins, overlapping by 1 °C while the 99.99^th^ percentile was calculated from a fitted generalized pareto distribution to the upper 1% of the data. As a reference, the grey dashed line represents the approximate Clausius-Clapeyron scaling of 7%. Vertical black lines represent the temperature range used in subsequent analysis. Figure was created using the statistical software ‘R’^61^.

Three regions are considered: North East America, Germany, and South East Australia as all these exhibit negative streamflow scaling but positive precipitation scaling. We focus on South East Australia (Fig. 2). For the 50^th^ percentile and 99^th^ percentile there is little indication that the streamflow scaling and precipitation scaling correspond. In particular for the 99^th^ percentile, if we consider the temperature range between 10 °C and 20 °C, the precipitation is increasing with higher temperature while the streamflow is notably decreasing. Only at the 99.99^th^ percentile does there appear to be a positive correlation between the precipitation-temperature trend and streamflow-temperature trend. Similar results are present for Germany and North East America (Figure S2). Despite the precipitation increasing with increasing temperature for each of the percentiles considered, only for the 99.99^th^ percentile does the streamflow increase. This suggests that when we are able to reduce the effect of precipitation losses, a correspondence between the streamflow and precipitation scaling ensues. From here on, we perform our analysis only on the temperature range highlighted, as outside this region the relationship with temperature can no longer be approximated as linear.

### Consideration of catchment size

**Figure 3:**
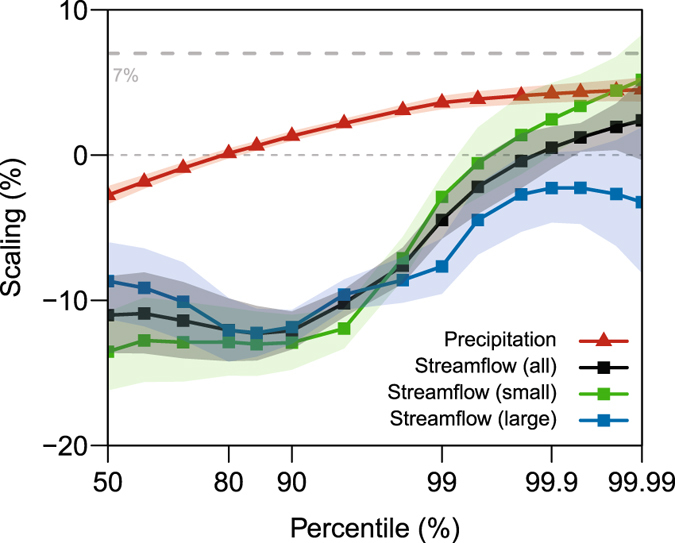
Precipitation and streamflow scaling with exceedance percentile demarcated on catchment area for South East Australia. Streamflow scaling for small catchments is in green, for large catchments in blue, and for all catchments in black. Precipitation scaling is in red. Shading represents 90% confidence limits. Scaling is calculated using linear regression on the percentiles for the temperature range 10 °C–20 °C as presented in Fig. 2. As a reference, the thick grey dashed line represents the approximate Clausius-Clapeyron scaling of 7%. Figure was created using the statistical software ‘R’^61^.

On average, the effects of precipitation losses are reduced with smaller catchment area^7^. For, if the catchment or region capturing the precipitation is smaller, but the precipitation intensity remains the same, the potential for losses is less. In large catchments, the peak streamflow is more likely to be influenced by the catchment wetness conditions preceding the storm event, whereas for smaller catchments, the streamflow is more likely to be a result of the precipitation which often causes flash flooding. Hence a greater correspondence between the precipitation and streamflow scaling should result for smaller catchments, and if hydrologic losses are to change with higher temperatures, larger catchments should be more sensitive to this change. We define small catchments as those smaller than 1 000 km^2^ and large catchments as those greater than 1 000 km^2^ as this demarcation splits our sample size approximately in half. The scaling for small (green), large (blue), and all (black) catchments as a function of exceedance percentile for South East Australia is presented in Fig. 3. The scaling has been calculated by fitting a linear regression for the temperature range of interest. The precipitation scaling is presented in red.

While our earlier results have shown streamflow scaling is consistently lower than precipitation scaling, for the small catchment case, streamflow scaling at the 99.99^th^ percentile now matches the scaling of the precipitation. This confirms that when the effect of precipitation losses is reduced, the precipitation and streamflow scaling tend to match, and indeed, it is changes in the ‘lost’ precipitation that are dominating the streamflow response to higher temperatures, particularly in larger catchments. The fact that corresponding results are found for NE America and Germany (Figure S3) which are climatologically different from the SE Australia suggests that these results are universal. A quantification of the change in precipitation not contributing to streamflow at higher temperatures is not attempted here due to the limited quality of data available on the initial catchment wetness. However, the above observation is consistent with other studies and knowledge that decreasing soil moisture wetness is associated with increasing temperatures^38, 39^.

### Consideration of transmission and anthropogenic effects

**Figure 4:**
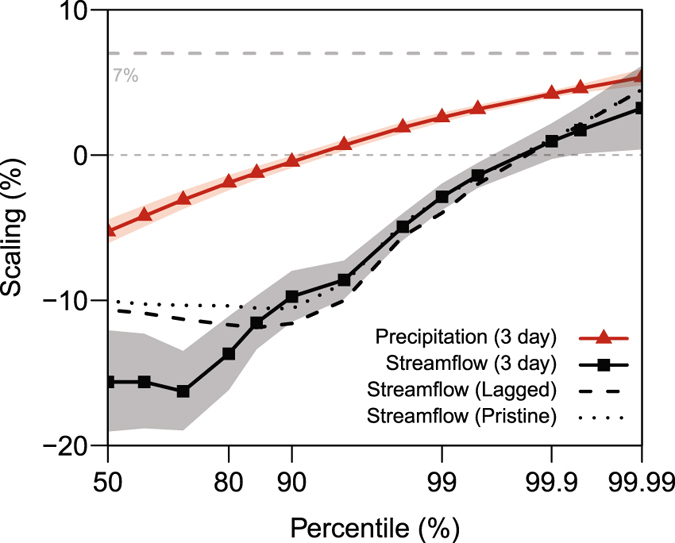
Sensitivity testing of streamflow scaling. Precipitation and streamflow scaling for three day accumulations is presented. Streamflow scaling is also presented using only pristine catchments and matched to the temperature corresponding to the streamflow inducing precipitation event, termed lagged. All streamflow scaling is presented in black, with precipitation scaling in red. Shading represents 90% confidence limits for the three day accumulation. Scaling is calculated using linear regression on the calculated percentiles as presented in Fig. 2. As a reference, the thick grey dashed line represents the approximate Clausius-Clapeyron scaling of 7%. Figure was created using the statistical software ‘R’^61^.

Although differing spatial scales have been considered, precipitation at the daily time scale may not correspond to recorded daily streamflow as the precipitation is attenuated as it travels through the catchment to the streamflow gauge. Further, it is also possible that the moisture source for a flood event does not have to coincide with the streamflow gauge due to advection of moisture^40^. Though aggregating spatially over homogenous regions is likely to reduce any effect of moisture advection on the sensitivities presented, in the absence of hydrologic modelling, a simple way to show temporal invariance is to aggregate the precipitation and streamflow to greater timescales. Figure 4 presents the scaling for three-daily precipitation and streamflow accumulations. The conclusions remain unchanged with the streamflow scaling falling below the precipitation scaling. The streamflow scaling remains negative below the 99.9^th^ percentile, and above this percentile approaches the precipitation scaling. It is also possible to consider durations shorter than daily, however, repeating the analysis using instantaneous maximum daily streamflow and hourly precipitation showed similar results.

There are additional complicating factors, such as anthropogenic effects which disrupt the catchment response, confounding the climatological temperature sensitivity. Urbanisation in recent years may have increased streamflow due to a larger proportion of impermeable surfaces^41^, while the development of dams for storage may have the opposite effect, reducing streamflow due to increased regulation^42^. A subset of the discharge database that is largely free of development and regulation and recommended by the World Meteorological Organisation to assess variability associated with climate variability was also adopted. This dataset, known as the climate sensitive data set, consists of catchments where less than 10% of the surface area has been modified and total extractions or regulations do not exceed 5% of the mean annual flow. In addition, all stations have continuous records over 20 years in length. It is not possible to get a perfect data set, but the correspondence of the scaling from this data (Fig. 4) to the results presented in Fig. 3 is encouraging. Finally, the temperature at which the flow is recorded does not necessarily correspond to the temperature which caused the storm event. A sensitivity test was performed using only streamflow peaks that had a preceding precipitation peak within seven days of the flow peak. The temperature corresponding to the precipitation peak (and not streamflow peak) was then matched to the streamflow. This ‘lagged’ time series (Fig. 4) does not substantially differ from the results presented in Fig. 3. Similar sensitivity tests are presented in Figure S4 for Germany and North East America. A lack of events means there is large uncertainty in the results, but, the overall conclusions do not change – only at the most extreme percentiles is there a correspondence between the rainfall and streamflow scaling.

## Discussion

Using the commonly applied criteria for extreme precipitation events of the 99^th^ percentile we found little evidence to suggest the generally positive scaling of precipitation in extratropical and temperate climates is replicated in streamflow. In fact, most regions scaled negatively implying a decrease in streamflow at higher temperatures. Even at the most extreme percentiles, where evidence of positive streamflow scaling was found, the scaling was less than the precipitation scaling. The only exception was for small catchments where the positive precipitation scaling matched the streamflow scaling. A similar result could be expected in urban catchments, where the effects of soil moisture and depression storage are more negligible.

As changes in precipitation are used to infer changes in flood response, the lack of correspondence between precipitation and streamflow scaling is concerning and suggests the sensitivity of precipitation to temperature is not a good indicator of interannual variability in streamflow peaks. The results presented here suggest that changes in ‘losses’ with higher temperatures, in particular, changes in soil moisture, play a large role in dictating changes to streamflow at higher temperatures. This is consistent with suggestions that if streamflow changes do not match precipitation changes, the most likely culprit is the initial moisture state of the catchment^43, 44^, and future research effort should focus on identifying trends and changes in the conditions preceding storm events in addition to changes in the storm event itself ^7, 45^. Quantification of the changes in each of the components in the water balance dictating catchment response was not attempted here due to the complicated nature of feedbacks and modelling complexity needed. For example, although higher temperatures are naturally associated with increases in evapotranspiration and reductions in soil moisture^46^, decreases in soil moisture have a positive feedback increasing surface temperatures, while also having a negative feedback, decreasing evapotranspiration^39, 47^. In addition to temperature, there are a number of other factors, such changes in atmospheric circulation patterns^29, 48^ and changes in the frequency and type of storm events^28, 35, 49^ that affect scaling relationships and are likely to impact streamflow in a future climate. Finally, changes in snowmelt have not been considered in this manuscript. Using precipitation and streamflow from the snowmelt free summer and autumn months showed little change to the results, suggesting snow does not affect the conclusions being drawn. However, it is important to comment that higher temperatures are expected to shift flooding to earlier in spring and increase the amount of precipitation in preference to snowfall^50^.

Based on the results presented, it can be concluded that a rise in temperature, while resulting in increased precipitation, results in an overall decrease in streamflow with the exception being rare to extreme precipitation events, or when the catchment is small, steep or impermeable. As a result, if historical sensitivities are continued to be used to evaluate future climate changes, it would be expected only the most extreme streamflow would increase, with the majority of streamflow events decreasing in a large part of the world.

## Methods

Daily precipitation was obtained from the Global Historical Climatology Network^51, 52^, daily streamflow from the Global Runoff Data Centre^53^, and daily gridded surface temperatures from Berkeley Earth^54–56^. Only stations operating for more than 10 years of record were used resulting in a total of 43121 precipitation stations and 5317 streamflow stations. Data was sampled as follows. First, precipitation events were identified. A precipitation event is defined here as one separated by five days of zero rain on either side. The maximum daily precipitation from each event was selected for analysis. Daily streamflow peaks for analysis were obtained by finding the maximum peak from streamflow events. Peaks were first identified, and then an event was defined if the peaks were separated by more than seven days. If they were, then the peak was adopted for analysis. If the peaks were separated by less than seven days only the maximum peak from the event was subsequently used. The different separation times ensured a similar number of events per year for the subsequent analysis. Each peak precipitation and streamflow observation chosen for analysis was matched to its coincident daily temperature. The temperature used was from the nearest 1° × 1° grid square to the site of interest. For precipitation this was the distance from the precipitation gauge to the centre of the grid square. For streamflow, it was the distance from the streamflow recording station (catchment outlet) to the centre of the grid square.

To help identify regions of homogenous scaling, Fig. 1 presents the scaling at each daily precipitation and streamflow site smoothed using a thin plate smoothing spline. Results at the gauge locations used in the smoothing are presented in Figure S1. The scaling was calculated using quantile regression on log transformed precipitation and streamflow^34^. As the precipitation-temperature sensitivity generally reverses at 24 °C due to moisture limitations at higher temepratures^24, 57^, for the regression calculation, the data was stratified on two temperature ranges, (1) 5–24 °C and (2) greater than 24 °C. Each site used precipitation and streamflow data for the temperature range which contained the majority of data. Streamflow and precipitation observed when the temperature was below 5 °C was ignored to remove the effects of snow melt.

For Figs 2 and S2 the point data in each region presented in Fig. 1 was aggregated to form a single data set. The data from each location was standardised by its mean before aggregation. Table S1 presents a summary of the streamflow record lengths while Table S2 presents a summary of the precipitation data length. The data was binned in 2 °C bins, overlapping by 1 °C, to reduce the effect of the arbitrary choice of bin boundaries^58^. For each bin, a generalized Pareto distribution was fit to the upper 1% of the data. The percentiles above 99^th^ percentile were then calculated from the fitted distribution^22^. Percentiles equal to and below the 99^th^ percentile were calculated empirically from the sample data. In Figs 3 and S3 the scaling is calculated by a linear regression to the log transformed precipitation and streamflow extreme percentiles for the temperature ranges shown in Figs 2 and S2. Due to the large sample sizes, it is unlikely that biases are introduced due to the bin boundary choice for the linear regression^34^. For Germany and SE Australia small catchments are defined as those less than 1 000 km^2^ in area and large catchments as those greater than 1 000 km^2^. To increase the sample size, For NE America a demarcation of 2 000 km^2^ is used, as there are very few catchments below 1 000 km^2^.

The analysis using all catchments was repeated by only sampling data from pristine catchments, using temperatures corresponding to the peak precipitation causing the streamflow event (termed lagged), and aggregating the precipitation and streamflow to three daily (Figs 4 and S4). In the case of the three day precipitation the duration of zero precipitation used to separate events was nine days. For streamflow, a duration of twenty-one days was used to separate peaks for analysis.

## Electronic supplementary material

Supplementary Information
