# Supplementary material for: Global assessment of flood and storm extremes with increased temperatures
Source: Sci Rep. 2017 Aug 11;7:7945. doi: 10.1038/s41598-017-08481-1 (PMC5554193; doi:10.1038/s41598-017-08481-1)
Supplement: Supplementary file 1 — Supplementary Information [file 41598_2017_8481_MOESM1_ESM.pdf]

Global assessment of flood and storm extremes with increased temperatures – Supplementary Information

Conrad Wasko[1] and Ashish Sharma[1]

1. School of Civil and Environmental Engineering, University of New South Wales, Sydney, Australia, 2052.

Table S1. Details of record lengths for streamflow data[1]

		Number of Stations	Median Record Length (Years)	Accumulated Record Length (Years)	Average Number of Events (per Year)
NE America	All	274 (119)	86	23020	14.8
	Small	87	77	6717	15.3
	Large	187	87	16303	14.6
Germany	All	317 (18)	64	22136	13.5
	Small	153	58	9290	13.2
	Large	158	74	12448	13.8
SE Australia	All	116 (34)	45	6061	14.9
	Small	69	42	3239	15.2
	Large	45	48	2685	14.6

[1] Number of pristine stations given in parenthesis.

Table S2. Details of record lengths for precipitation data

	Number of Stations	Median Record Length (Years)	Accumulated Record Length (Years)	Average Number of Events (per Year)
NE America	6333	40	4726667	15.2
Germany	478	65	399567	13.3
SE Australia	4349	45	3919329	16.1

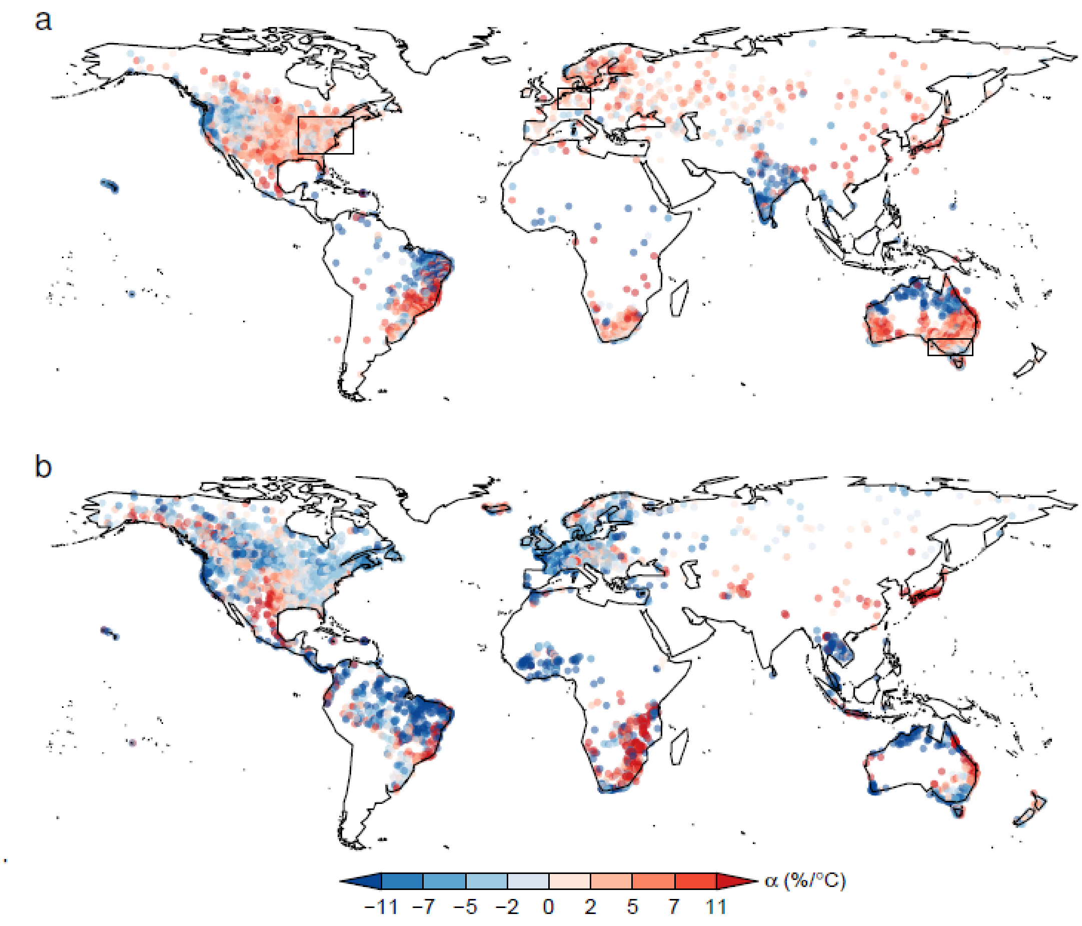

Figure S1. Precipitation and streamflow scaling with temperature for the 99th percentile using quantile regression. (a) Precipitation scaling. (b) Streamflow scaling. Figure was created using the 'maps'[59] and 'mapdata'[60] packages in the statistical software 'R'[61].

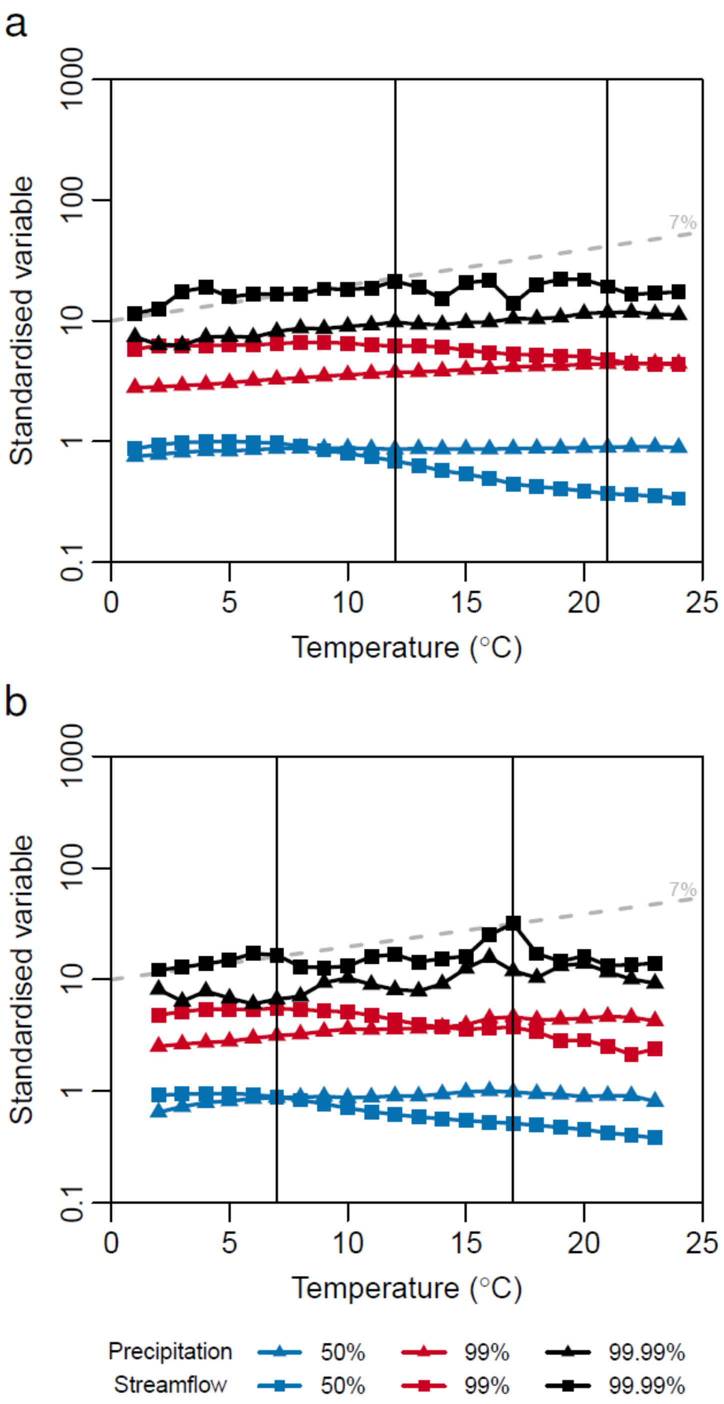

Figure S2. Percentiles of precipitation and streamflow with temperature. (a) North East America (b) Germany. The 50th and 99th percentile were calculated empirically from the data using 2°C temperature bins, overlapping by 1°C while the 99.99th percentile was calculated from a fitted generalized pareto distribution to the upper 1% of the data. As a reference, the grey dashed line represents the approximate Clausius-Clapeyron scaling of 7%. Vertical black lines represent the temperature range used in subsequent analysis. Figure was created using the statistical software 'R'[61].

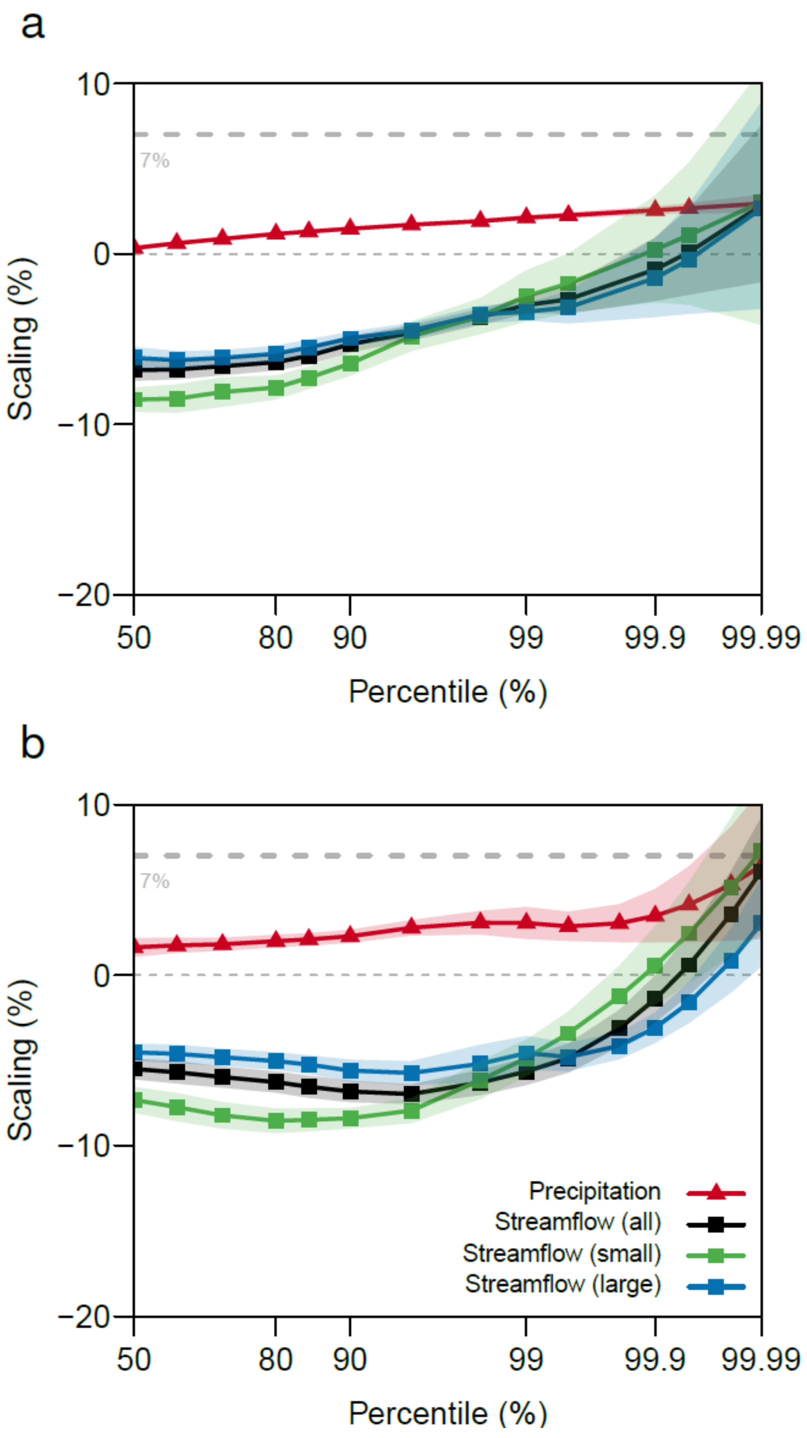

Figure S3. Precipitation and streamflow scaling with exceedance percentile demarcated on catchment area. (a) North East America (b) Germany. Scaling for small catchments is in green, scaling for large catchments is in blue. Scaling using all catchments is in black. Precipitation scaling is in red. Shading represents 90% confidence limits. Scaling is calculated using linear regression on the percentiles for the temperature ranges presented in Figure S2. As a reference, the thick grey dashed line represents the approximate Clausius-Clapeyron scaling of 7%. Figure was created using the statistical software 'R'[61].

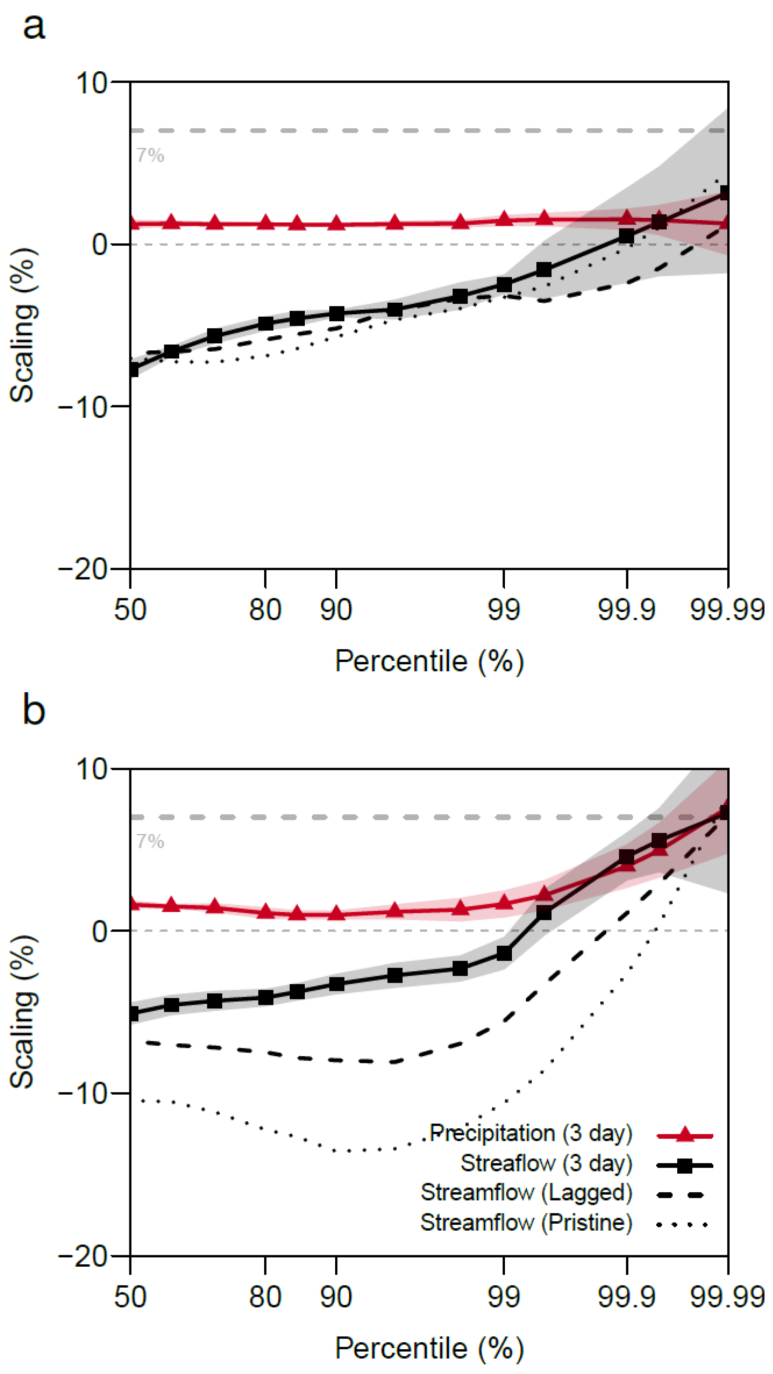

Figure S4. Sensitivity testing of streamflow scaling. (a) North East America (b) Germany. Three day accumulations for precipitation and streamflow scaling are presented. Streamflow scaling is also presented using only pristine catchments and matched to the temperature corresponding to the streamflow inducing precipitation event. All streamflow scaling is presented in black, with precipitation scaling in red. Shading represents 90% confidence limits. Scaling is calculated using linear regression on the calculated percentiles as presented in Figure S2. As a reference, the thick grey dashed line represents the approximate Clausius-Clapeyron scaling of 7%. Figure was created using the statistical software 'R'[61].
